# Seasonal flooded rice area extent dataset during dry seasons 2016 to 2019 at the Telangana state scale, South-India

**DOI:** 10.1016/j.dib.2025.111981

**Published:** 2025-08-11

**Authors:** Sylvain Ferrant, Adrien Selles, Arthur Vincent, Vincent Thierion, Olivier Hagolle, Ahmed Shakeel, Virendra M. Tiwari

**Affiliations:** a CESBIO, Université de Toulouse, CNES/CNRS/INRAE/IRD/UT3, 18 avenue Edouard Belin BPI 2801, 31401 Toulouse Cedex 9, France; b BRGM, Université de Montpellier, Montpellier, France, G-Eau, INRAE, CIRAD, IRD, AgroParisTech, Institut Agro, 1039 Rue de Pinville 34000, Montpellier, France; c CS group, 6, rue Brindejonc des Moulinais BP 15872, 31506 Toulouse Cedex 5, France; d CNES, 18 Av. Edouard Belin, 31401 Toulouse cedex 9, France; e CSIR-National Geophysical Research Institute, CH82+G9Q, Uppal Rd, NGRI, Habsiguda, Hyderabad, Telangana 500007, India; f Islamic University of Science and Technology, Administrative Block IUST, IUST, University Ave, Awantipora, 192122, India; g CSIR - North East Institute of Science & Technology, P5Q4+F98, NH-37, Pulibor, Jorhat, Sensowa Gaon, Assam 785006, India

**Keywords:** Irrigated crop classifications, Orfeo ToolBox, IOTA2, Sentinel-2, South-Asia, Time series interpolation, Field validation, Agricultural statistics

## Abstract

Indian agriculture largely depends on the timely and spatially variable availability of water resources which are replenished during the monsoon season. In the state of Telangana, a significant portion of the available water is utilized for flooded rice cultivation, both in surface water-fed command areas and in groundwater-dependent regions. The spatial extent of seasonal rice cultivation varies annually in response to water availability that is a key indicator of how farmers adapt to regional and global environmental and socio-economic changes. In this study, we present seasonal land use maps for the dry season (Rabi) from 2016 to 2019, derived using the *Infrastructure pour l'Occupation des sols par Traitement Automatique* (IOTA²) processing chain [1]. IOTA² is an open-source software that combines temporal interpolation and classification of multispectral Sentinel-2 time series to map land cover dynamics. Sentinel-2 Level 2A data—processed using the Multi-Temporal Cloud Screening and Atmospheric Correction Software (MAJA)—were used to generate 10-day composite reflectance time series for each season over the entire Telangana state. A Random Forest classifier was trained on interpolated spectral time series using ground-truth data collected by the authors during dedicated field campaigns conducted between January and March of each year from 2016 to 2019. Ground observations were labelled into nine land use classes: rice, vegetables, maize (when applicable), orchards, natural bush, bare ground, urban, water, and unharvested dry-season cotton (when applicable). For each season, the ground-truth dataset was randomly split into training and validation sets eight times to generate eight classification outputs, from which average precision, recall, and F-score values were calculated. The dataset associated with this paper includes four seasonal raster maps, each encoding, for every pixel, the number of times (from 0 to 8) it was classified as rice during the eight classification runs. These rice extent confidence maps serve as an empirical measure of spatial classification uncertainty and inter-annual variability. The ground-truth polygon dataset used for classification and validation is also provided. Together, these datasets support the monitoring of seasonal rice dynamics and can serve as a reference for agricultural and hydrological studies in South Asia or training data for deep learning approaches for extension in space and time of those maps. Such a compilation can be used to support decisions on crop or cropping pattern changes in response to climate change, as well as to inform government policy-making.

Specifications Table

**Table:** 

Subject	Agronomy and Crop Science
Specific subject area	*Sentinel-2 derived dry season rice mapping using 8 random forest classifications from a set of crop cover surveys field trip in the Indian Telangana state between 2016 and 2019.*
Type of data	Four GeoTIFF files (integer values ranging from 0 to 8) covering the border areas of Telangana State at a 10-meter resolution. These files indicate the number of times a pixel was classified as rice across eight Random Forest classification runs for the given growing seasons (January to April in 2016, 2017, 2018, and 2019). Each of these eight classifications was generated using a different random selection of training samples from the available ground-truth dataset. GeoTIFF Image Four Shapefile of polygones per season containing the ground truth, i.e. the type of crop observed during the four season of field surveys
Data collection	To collect ground-truth data, a car were used across the Telangana state for each growing season with a laptop running QGIS [3], displaying the most recent Sentinel-2 image. A BU-353S4 USB GPS receiver provided real-time vehicle location, allowing the operator to
	delineate field boundaries for various crop types within the area of interest, with a particular focus on rice fields. Only identifiable crops over large enough fields (>100m) were selected to avoid mixels: mixed 10m*10m Sentinel-2 pixels covering various crops. The final product consists of a shapefile for each season, containing the outlines of pure pixels—those represents only a single crop type—crop type being largely found within the study region (Rice, Vegetables, irrigated corn, bareground, trees etc...
Data source location	The data set is located within the Telangana state, south India. Rasters are stored here: https://datasuds-geo.ird.fr/geonetwork/umr_cesbio/eng/catalog.search#/metadata/1f7bb3d2-4223-4e1d-a39d-7e432bcbbb34 Vectors are stored here: https://datasuds-geo.ird.fr/geonetwork/umr_cesbio/eng/catalog.search#/metadata/dab0b558-ef5f-4df1-ba7c-714dff15b6ec
Data accessibility	Repository name: DataSuds-geo.ird.fr Data identification number: https://doi.org/10.23708/1f7bb3d2-4223-4e1d-a39d-7e432bcbbb34 https://doi.org/10.23708/dab0b558-ef5f-4df1-ba7c-714dff15b6ec Direct URL to data: https://datasuds-geo.ird.fr/geonetwork/umr_cesbio/eng/catalog.search#/metadata/1f7bb3d2-4223-4e1d-a39d-7e432bcbbb34https://datasuds-geo.ird.fr/geonetwork/umr_cesbio/eng/catalog.search#/metadata/dab0b558-ef5f-4df1-ba7c-714dff15b6ec Two types of formats: online streaming (WMS) and direct download (geotif for rasters and a choice of vector format for vector layers)
Related research article	Pascal, C.; Ferrant, S.; Rodriguez-Fernandez, N.; Kerr, Y.H.; Selles, A.; Merlin, O. “Indicator of Flood-Irrigated Crops from SMOS and SMAP Soil Moisture Products in Southern India,” in IEEE Geoscience and Remote Sensing Letters, vol. 20, pp. 1-5, 2023, Art no. 4500205, https://doi.org/10.1109/LGRS.2023.3267825

## 1 Value of the Data

• Producing these high-resolution rice maps requires handling terabytes of satellite data, addressing cloud contamination, radiometric correction and interpolating reflectance values, and generating multi-temporal cubes to extract rice classes. This process, conducted at a regional scale using a high-performance computing cluster, is beyond the reach of many researchers due to download speed, computational resources and expertise involved. Furthermore, the data come with an uncertainty stemming from the random sampling process used in the training data, with eight different random draws generating eight distinct rice maps, each with associated variability in classification accuracy, left at the user appreciation.
• These data enable researchers to study the evolution of rice cultivation in India, particularly in response to climatic variability and infrastructure development aimed at improving irrigation. Unlike government statistics, which are aggregated at the Mandal level (A district sub-division) and not always consistent between years, these maps provide high-resolution (10m) spatial insights into seasonal rice distribution.
• At 10m resolution, these maps enable the detection of rice fields scattered across the landscape, particularly in upstream areas where rice cultivation represents only a small percentage of the land cover. Such fine-scale detection is crucial for identifying fragmented or low-density rice-growing regions that coarser-resolution products (e.g., 500m or 300m) fail to capture. This improved spatial granularity provides a more accurate representation of rice extent, benefiting studies on water resource management, land-use planning, and agricultural policy.
• The dataset can be used to test and compare various classification approaches, including Convolutional Neural Networks (CNNs) and unsupervised learning methods. Researchers can assess how different techniques perform on high-resolution Sentinel-2 imagery and explore potential improvements in rice mapping.
• The methodology and data can be extended to other regions or years, facilitating broader studies on rice expansion, land-use change, and food security. The dataset serves as a reference for transferring models beyond Telangana.
• These data hold value for multiple disciplines, including remote sensing, agronomy, hydrology and hydro-geology, economics, and climate studies. They allow economists to refine agricultural productivity assessments beyond traditional government statistics.

## 2 Background

High-resolution (10m) seasonal rice maps are crucial to understanding the Telangana's agricultural dynamics strongly influenced by water availability and irrigation infrastructures. While recent efforts have aimed to produce seasonal rice extent maps at the national level using Sentinel-1 radar data without publishing the maps [4], these approaches face limitations in regions like Telangana. The predominantly groundwater-fed irrigation systems and scattered planting patterns around bore-wells make it difficult for Sentinel-1 data to reliably detect rice fields [5]. Furthermore, although these national-scale maps exist for 2018 and 2020, the underlying datasets are not yet publicly available. In contrast, optical data from Sentinel-2 provide higher spatial and spectral resolution, offering a better alternative for mapping small, fragmented rice fields during the dry, cloud free seasons. This study was possible at the state level thanks to the production of Sentinel-2 time series with the MAJA atmospheric correction algorithm [2] operated by Theia Data and Services centre for continental surfaces and the use of IOTA² classification framework (developed in CESBIO). Seasonal field campaigns in Telangana have been hosted by the National Geophysical Research Institute in Hyderabad, thanks to the fruitful collaboration established at the time. Field data were used to train and validate the classifier. These maps have also supported broader research into seasonal soil moisture dynamics as observed by passive microwave sensors such as SMOS and SMAP, which operate at a coarser spatial resolution (25 km) [3,4].

## 3 Data Description

The raster dataset found in this collection:

https://datasuds-geo.ird.fr/geonetwork/umr_cesbio/eng/catalog.search#/metadata/1f7bb3d2-4223-4e1d-a39d-7e432bcbbb34 is made by 4 seasonal datasets available in WMS and GEOTIF named as follows:

RGAE_Rabi_10m_JanApr2016_RF8samples for the Rice Growing Area Extent at 10 meters resolution for the dry season from January to April 2016, corresponding to a 10 meter raster resolution with numerical values (from 0 to 8) that correspond to the number of times the pixel has been classified as rice.

The vector dataset found in this collection:

https://datasuds-geo.ird.fr/geonetwork/umr_cesbio/eng/catalog.search#/metadata/dab0b558-ef5f-4df1-ba7c-714dff15b6ec

Is made by 4 seasonal field survey vectors available for downloading in WFS, or online streaming in WMS. Each vector file corresponds to a field survey made in March of each year (2016 to 2019), with a focus on rice but also other landcover. Each polygon has an ID number corresponding to the type of landcover: 1- Rice; 2- Vegetables; 3- Maize; 4- Orchard; 5- Natural bush/forest; 6- Bare-ground; 7- Urban; -8 Water; 9- Non harvested dry Cotton (when applicable). Urban and natural areas, being relatively stable during the study period, were extracted from the 10 m WorldCover dataset. In contrast, dynamic classes like water, bare soil and crops were manually sampled due to their temporal variability. Maize and vegetables were occasionally recorded in the field data but were absent in some years. Cotton, a rainfed crop grown during the monsoon season, was sometimes still present as unharvested dry biomass during the dry season; this class was therefore only observed in certain years.

## 4 Experimental Design, Materials and Methods

Cluster based classification pipeline:

This study involves the production of seasonal rice maps from optical Sentinel-2 data for the dry season only, from January to April, spanning the years 2016 to 2019. The Indian monsoon season (July–September) is characterized by near-continuous cloud cover, which makes optical remote sensing not feasible for mapping rice during this period. While radar data such as Sentinel-1 can penetrate clouds, expected classification accuracy remain lower due to coarser resolutions [4,5]. Sentinel-2 Level-1C imagery were, acquired through the Copernicus program, were used to produce surface reflectance products generated using the MAJA atmospheric correction algorithm (https://github.com/CNES/MAJA), developed by CNES French spatial agency and CESBIO laboratory [2]. The data are processed and distributed by the Theia Land Data Centre (https://www.theia-land.fr/en), a French platform that provides value-added Earth observation products for environmental monitoring (now https://geodes-portal.cnes.fr/). The Level-2A products were downloaded from Theia’s repository onto the CNES TREX computing cluster (https://cnes.fr/en/projects/centre-de-calcul), where the IOTA² processing chain [1] was subsequently applied (https://framagit.org/iota2-project/iota2). This processing chain starts with an interpolation of each Sentinel-2 bands over time at a 10-day temporal resolution during the dry season (December to April) for each growing season for the years 2016 to 2019. The study area spans 28 Sentinel-2 tiles over the Telangana state in South India, covering the following tile IDs: 43PGT 43PHT 43QFA 43QFU 43QFV 43QGA 43QGB 43QGU 43QGV 43QHA 43QHB 43QHU 43QHV 44PKC 44QKD 44QKE 44QKF 44QKG 44QLD 44QLE 44QLF 44QLG 44QMD 44QME 44QMF 44QMG 44QNE 44QNF.

**Fig. 1:**
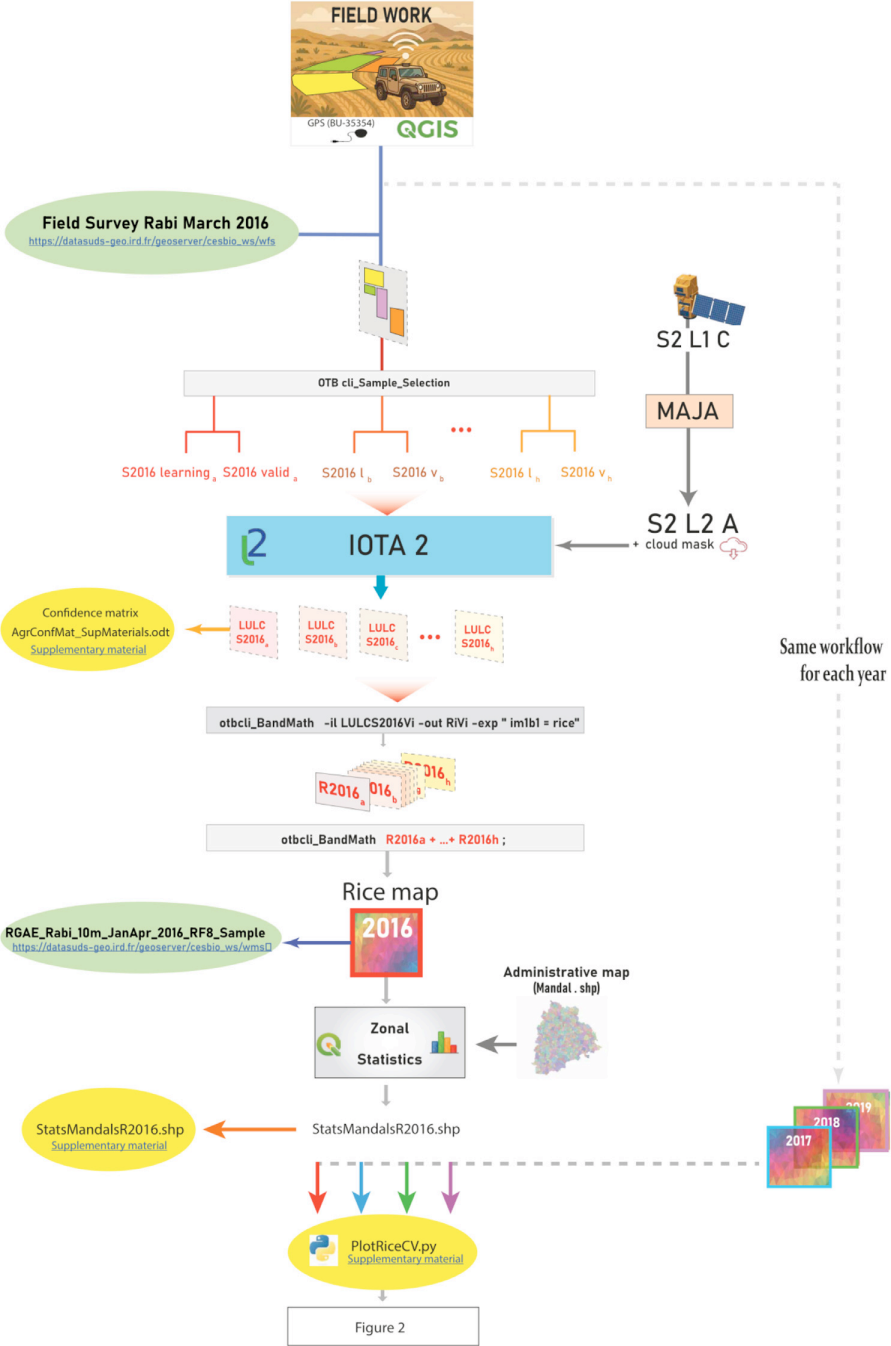
Flowsheet illustrating the data processing pipeline, from raw input acquisition to the generation of final published output products and supplementary materials.

The processing chain allows also to perform random selections of field samples to produce several classification of the same crop growing season. For each of the four dry seasons, 8 random sampling iterations were performed to create learning datasets. These samples were used successively to train the Random Forest classifier. From the 8 classification produced, the number of times each Sentinel-2 pixel was classified as rice was counted using BandMath from Orfeo Tool Box, an equivalent of QGIS raster calculator. This process resulted in a map for each season, where the value of each pixel ranges from 0 to 8, indicating the number of times the pixel was classified as rice across the 8 iterations. The whole work is submarized in the Fig. 1 under the form of a flowsheet.

The final published data consists of 4 raster datasets, one for each season, with values representing the classification frequency of rice pixels across the entire Telangana region. These maps provide valuable insights into the inter-annual dynamics of rice cultivation in the study area. All processing steps, including data pre-processing, classification, and validation, were carried out using open-source tools and software available within the IOTA2 framework which is based on Orfeo ToolBox libraries [6] (https://www.orfeo-toolbox.org/).

Field surveys:

Field data collection was conducted through road surveys across the study area. A laptop equipped with QGIS was used in the vehicle, displaying the most recent Sentinel-2 imagery (including Normalized Difference Vegetation Index (NDVI) and Normalized Difference Wetness Index (NDWI) composites) to help identify irrigated fields, active cropping areas, or fallow lands. A BU-353S4 GPS receiver (GlobalSat), magnetically attached to the vehicle roof, provided real-time geolocation within QGIS (menu view➔Toolbars➔GPS). Crop and land cover polygons were manually digitized during the survey, focusing on dynamic and seasonally varying land use types.

In addition to the field surveys, samples for more stable land cover classes—such as urban areas, forests, and permanent water bodies—were later supplemented at the office using expert interpretation based on Sentinel-2 time series, Bing aerial imagery, and the WorldCover 10 m product. These digitized vector datasets were then used as the reference database for generating eight random training/testing sample draws per season for classification (step included in IOTA2 chain).

Validation strategies:

Aggregated confusion matrices (mean values and standard deviations over the 8 random sampling iterations) for each dry season from January to April 2016–2019 are provided in the supplementary material. These results confirm the very high classification accuracy of the rice class, with F-scores systematically above 0.98 across all seasons, and very limited confusion with other land cover types such as orchards, natural bush, or water. This strong consistency justifies the use of these maps for spatial analyses focused on irrigated rice cropping. Other classes were not the target of this study, as they either represent non-irrigated systems or land covers with marginal water use during the dry season.

**Fig. 2:**
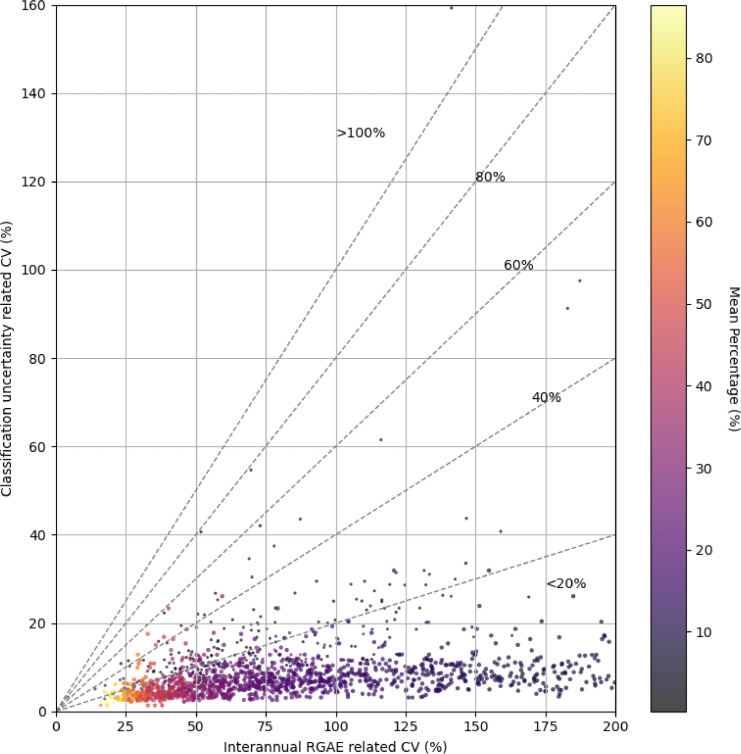
Relationship between inter-annual variability of rice crop area percentages and classification uncertainty at the Mandal scale in Telangana (India), for the dry seasons of 2016 to 2019. Each dot represents a Mandal (district sub-division), with color indicating the mean rice area percentage across the four seasons (2016–2019).

Despite these excellent classification metrics for the rice class across all seasons, the estimated proportion of rice within each Mandal (A district sub-division) can vary significantly between the 8 random sampling iterations, especially in areas with intermediate or low rice coverage. This intra-seasonal uncertainty, stemming from the sampling-induced variability in training data, can sometimes approach or even exceed the inter-annual variability of rice extent. Fig. 2 illustrates this relationship by comparing, for each Mandal, the coefficient of variation (CV) of rice percentages due to classification uncertainty (i.e., across the 8 sampling iterations) with the inter-annual CV (i.e., over the four dry seasons). The colour scale represents the average rice coverage. This visualization highlights that in several Mandals, classification uncertainty remains substantial, underscoring the need to account for this variability when interpreting rice area trends or validating against agricultural statistics. The python code and shapefiles used for creating this figure can be found in supplemental materials.

Each shapefile provided in the supplemental material corresponds to one dry season (from 2016 to 2019) and contains rice statistics at the Mandal level. For each season, the shapefile includes eight columns named Rice_pct_1 to rice_pct_8, which represent the percentage of rice pixels per Mandal obtained from eight classification runs (seeds). These values were computed using the “Zonal Statistics” algorithm in QGIS (version 3.16.15), which aggregates pixel-level classification results into administrative boundaries. The Mandal boundaries were derived from the publicly available GeoJSON file TELANGANA_SUBDISTRICTS.geojson provided on a public GitHub repository (https://github.com/datta07/INDIAN-SHAPEFILES). These seasonal shapefiles are used by the accompanying Python scripts (PlotRiceCV.py) to calculate the coefficient of variation (CV) per Mandal and generate the boxplots shown in Figure X.

## Limitations

The mapping of dry-season rice in India using Sentinel-2 imagery is subject to several limitations. While classification uncertainty is explicitly assessed through the generation of eight maps per season using different random draws of training data, and confusion matrices are provided (see supplemental material), additional sources of error remain. Cloud cover reduces observation density unevenly across space and time, and local variations in sowing dates or irrigation access can disrupt phenological signals. Furthermore, ground-truth data were collected over several years (2016–2019), introducing temporal inconsistencies that may affect model generalization.

Rice area estimates are extracted from a land cover classification with high overall accuracy, but class confusion exists—particularly between bare ground, natural vegetation, and urban areas—due to weaker sampling for non-rice classes and class imbalance. Nevertheless, the rice class is consistently well identified, with F-scores above 0.98, thanks to its distinctive temporal signature during the dry season. These results are much higher than those obtained from Sentinel-1 in Telangana state reported in [4].

Despite these challenges, the ensemble classification approach mitigates sampling-induced uncertainty. Fig. 2 illustrates how classification variability (CV across the 8 samplings) relates to inter-annual variability and mean rice coverage, showing that uncertainty remains moderate and acceptable when compared to typical errors in agricultural statistics and the spatial heterogeneity of smallholder rice systems. This dataset was used in previous studies to relate observed rise of soil water content from Soil Moisture and Ocean Salinity (SMOS) and Soil Moisture Active and Passive (SMAP) satellite Soil Moisture(SM) products during the dry season. This variable is directly proportional to the seasonal rice area extent within the SM product pixel (around 25 km) [7,8].

## Ethics Statement

The authors have read and follow the ethical requirements for publication in Data in Brief and confirming that the current work does not involve human subjects, animal experiments, or any data collected from social media platforms.

## CRediT author statement

Sylvain Ferrant conceptualized and designed the study, established the field data collection protocol, and actively participated in several field campaigns. He coordinated the processing of Sentinel-2 data on the CNES cluster, played a leading role in mobilizing the Theia consortium to produce large-scale L2A data, and led the writing, figure production, and final editing of the manuscript.

Adrien Selles was instrumental in enabling and organizing all fieldwork in India. He managed local logistics, administrative procedures, and permission processes, ensuring the success of the ground campaigns. He also contributed to the review and refinement of the manuscript.

Arthur Vincent and Vincent Thierion provided indispensable technical support for the implementation of the IOTA2 classification chain and cluster computing. They were key to the validation and interpretation of classification results. Vincent Thierion also assisted in the creation of several figures.

Olivier Hagolle played a crucial strategic role in the Sentinel-2 L2A data production effort through the Theia consortium. His vision and long-term support were essential to making these data publicly available and useful for scientific applications.

Ahmed Shakeel and Virendra M. Tiwari ensured the long-term viability of this research through their institutional and scientific support at NGRI. Their collaboration and coordination allowed repeated fieldwork campaigns to take place over several years. They have also contributed in the finalization of the manuscript.

## Data Availability

datasuds-geo.ird.frSeasonnal Rice Growing Area Extent in Telangana (South-India) - Vector data (Original data)
datasuds-geo.ird.frSeasonnal Rice Growing Area Extent in Telangana state (South-India) - Raster data (Original data)

## Appendix Supplementary materials

Image, application 1

Image, application 2
