# Supplementary material for: Seasonal flooded rice area extent dataset during dry seasons 2016 to 2019 at the Telangana state scale, South-India
Source: Data Brief. 2025 Aug 11;62:111981. doi: 10.1016/j.dib.2025.111981 (PMC12396292; doi:10.1016/j.dib.2025.111981)
Supplement: Supplementary file 1 [file mmc1.zip › AgrConfMat_SupMaterials.odt]

Jan. – Apr. 2016

KAPPA : 0.867 ± 0.020
Overall Accuracy (OA) : 0.902 ± 0.017

| Classes | Precision mean | Recall mean | F-score mean | Main Confusions |
| --- | --- | --- | --- | --- |
| Rice | 0.985 ± 0.008 | 0.982 ± 0.004 | 0.983 ± 0.004 | Rice, Orchards, Natural Bush |
| Vegetables | 0.000 ± nan | 0.000 ± nan | 0.000 ± nan | Natural Bush, Orchards, bareground |
| Maize | 0.000 ± nan | 0.000 ± nan | 0.000 ± nan | Natural Bush, Rice, Orchards |
| Orchards | 0.889 ± 0.033 | 0.816 ± 0.052 | 0.848 ± 0.026 | Orchards, Natural Bush, Urban |
| Natural Bush | 0.790 ± 0.045 | 0.962 ± 0.029 | 0.866 ± 0.032 | Natural Bush, Orchards, bareground |
| bareground | 0.665 ± 0.191 | 0.486 ± 0.188 | 0.552 ± 0.188 | bareground, Natural Bush, Rainfed Cotton unharvested (when applicable) |
| Urban | 0.805 ± 0.126 | 0.693 ± 0.127 | 0.719 ± 0.078 | Urban, Natural Bush, bareground |
| Water | 1.000 ± 0.000 | 0.999 ± 0.001 | 0.999 ± 0.001 | Water, Rice, Orchards |
| Rainfed Cotton unharvested (if applicable) | 0.875 ± 0.065 | 0.673 ± 0.093 | 0.753 ± 0.063 | Rainfed Cotton unharvested, Natural Bush, bareground |

Jan.-Apr. 2017

KAPPA : 0.848 ± 0.029
Overall Accuracy (OA) : 0.890 ± 0.021

| Classes | Precision mean | Recall mean | F-score mean | Main Confusions |
| --- | --- | --- | --- | --- |
| Rice | 0.989 ± 0.008 | 0.988 ± 0.003 | 0.988 ± 0.004 | Rice, Orchards, Natural Bush |
| Vegetables | 0.000 ± nan | 0.000 ± nan | 0.000 ± nan | Natural Bush, Orchards, Rice |
| Maize | 0.477 ± 0.417 | 0.104 ± 0.124 | 0.160 ± 0.185 | Natural Bush, Rice, Maize |
| Orchards | 0.801 ± 0.103 | 0.833 ± 0.050 | 0.807 ± 0.036 | Orchards, Natural Bush, Rice |
| Natural Bush | 0.856 ± 0.030 | 0.909 ± 0.058 | 0.880 ± 0.027 | Natural Bush, Orchards, bareground |
| bareground | 0.643 ± 0.237 | 0.588 ± 0.220 | 0.524 ± 0.191 | bareground, Natural Bush, Urban |
| Urban | 0.968 ± 0.020 | 0.843 ± 0.017 | 0.901 ± 0.012 | Urban, Natural Bush, Orchards |
| Water | 1.000 ± nan | 0.995 ± 0.005 | 0.998 ± 0.003 | Water, Rice, Orchards |

Jan.-Apr. 2018

KAPPA : 0.949 ± 0.007
Overall Accuracy (OA) : 0.962 ± 0.006

| Classes | Precision mean | Recall mean | F-score mean | Main Confusions |
| --- | --- | --- | --- | --- |
| Rice | 0.986 ± 0.003 | 0.985 ± 0.004 | 0.986 ± 0.001 | Rice, Orchards, Natural Bush |
| Vegetables | 0.000 ± nan | 0.000 ± nan | 0.000 ± nan | Orchards, Natural Bush, Rice |
| Maize | 0.514 ± 0.411 | 0.040 ± 0.070 | 0.067 ± 0.109 | Rice, Natural Bush, Orchards |
| Orchards | 0.875 ± 0.033 | 0.834 ± 0.037 | 0.853 ± 0.025 | Orchards, Natural Bush, Urban |
| Natural Bush | 0.927 ± 0.020 | 0.979 ± 0.011 | 0.952 ± 0.013 | Natural Bush, Orchards, Urban |
| bareground | 0.831 ± 0.058 | 0.754 ± 0.037 | 0.789 ± 0.036 | bareground, Natural Bush, Urban |
| Urban | 0.980 ± 0.010 | 0.935 ± 0.013 | 0.957 ± 0.010 | Urban, Orchards, Natural Bush |
| Water | 1.000 ± 0.000 | 0.999 ± 0.002 | 0.999 ± 0.001 | Water, Natural Bush, Orchards |

Jan.-Apr. 2019

KAPPA : 0.962 ± 0.008
Overall Accuracy (OA) : 0.975 ± 0.005

| Classes | Precision mean | Recall mean | F-score mean | Main Confusions |
| --- | --- | --- | --- | --- |
| Rice | 0.981 ± 0.005 | 0.985 ± 0.003 | 0.983 ± 0.003 | Rice, Urban, Vegetables |
| Vegetables | 0.107 ± 0.110 | 0.038 ± 0.039 | 0.055 ± 0.056 | Rice, bareground, Urban |
| Maize | 0.000 ± nan | 0.000 ± nan | 0.000 ± nan | Rice, Urban, bareground |
| Natural Bush | 0.000 ± nan | 0.000 ± nan | 0.000 ± nan | bareground, Rice, Water |
| bareground | 0.687 ± 0.034 | 0.811 ± 0.065 | 0.742 ± 0.038 | bareground, Urban, Rice |
| Urban | 0.973 ± 0.009 | 0.991 ± 0.002 | 0.982 ± 0.005 | Urban, bareground, Rice |
| Water | 1.000 ± 0.000 | 0.999 ± 0.001 | 0.999 ± 0.001 | Water, Rice, bareground |
| Rainfed Cotton unharvested | 0.067 ± 0.140 | 0.010 ± 0.021 | 0.017 ± 0.036 | bareground, Urban, Rice |
